# Supplementary material for: Long-term monitoring of intravital biological processes using fluorescent protein-assisted NIR-II imaging
Source: Nat Commun. 2022 Nov 4;13:6643. doi: 10.1038/s41467-022-34274-w (PMC9636246; doi:10.1038/s41467-022-34274-w)
Supplement: Supplementary file 1 — Supplementary Information [file 41467_2022_34274_MOESM1_ESM.pdf]

Supplementary Information:

Long-term monitoring of intravital biological processes using fluorescent protein-assisted NIR-II imaging

Chen et al

Long-term monitoring of intravital biological processes using fluorescent protein-assisted NIR-II imaging

Supplementary Figure 1. iRFPs purification.

Supplementary Figure 2. NIR-II fluorescence imaging of stable iRFP713-expressing cells.

Supplementary Figure 3. NIR-II fluorescence imaging of mice infected with iRFP713-encoding adenovirus.

Supplementary Figure 4. Construction of iRFP713 transgenic reporter mice.

Supplementary Figure 5. The expression pattern of iRFP713 in different tissues of iRFP713 general expression mice.

Supplementary Figure 6. Specific expression of iRFP713 in the liver of hepatocyte-specific iRFP713 expressing mice.

Supplementary Figure 7. Liver morphology of the hepatocyte-specific iRFP713 expression mice after PHX.

Supplementary Figure 8. Effect of iRFP713 expression on hepatocyte proliferation after PHX.

Supplementary Figure 9. Hepatotoxicity and NIR-II fluorescence *in vivo* imaging of ICG.

Supplementary Figure 10. Comparation of liver proliferation and injury between iRFP713 and ICG.

Supplementary Figure 11. Characterization of APAP toxicity model.

Supplementary Figure 12. Liver injury and recovery of hepatocyte-specific iRFP713 expressing mice treated with APAP.

Supplementary Figure 13. Characterization of multiple low-dose STZ induced diabetes.

Supplementary Figure 14. Long-term expression stability of iRFP713 transgenic mice.

Supplementary Figure 15. Comparison of iRFP713 imaging in NIR-I and NIR-II regions.

Supplementary Table 1. The calculated area SBR values.

Supplementary Table 2. Primers of iRFP713.

Supplementary Table 3. Antibodies used for experiments.

Supplementary Figure 1. iRFPs purification.

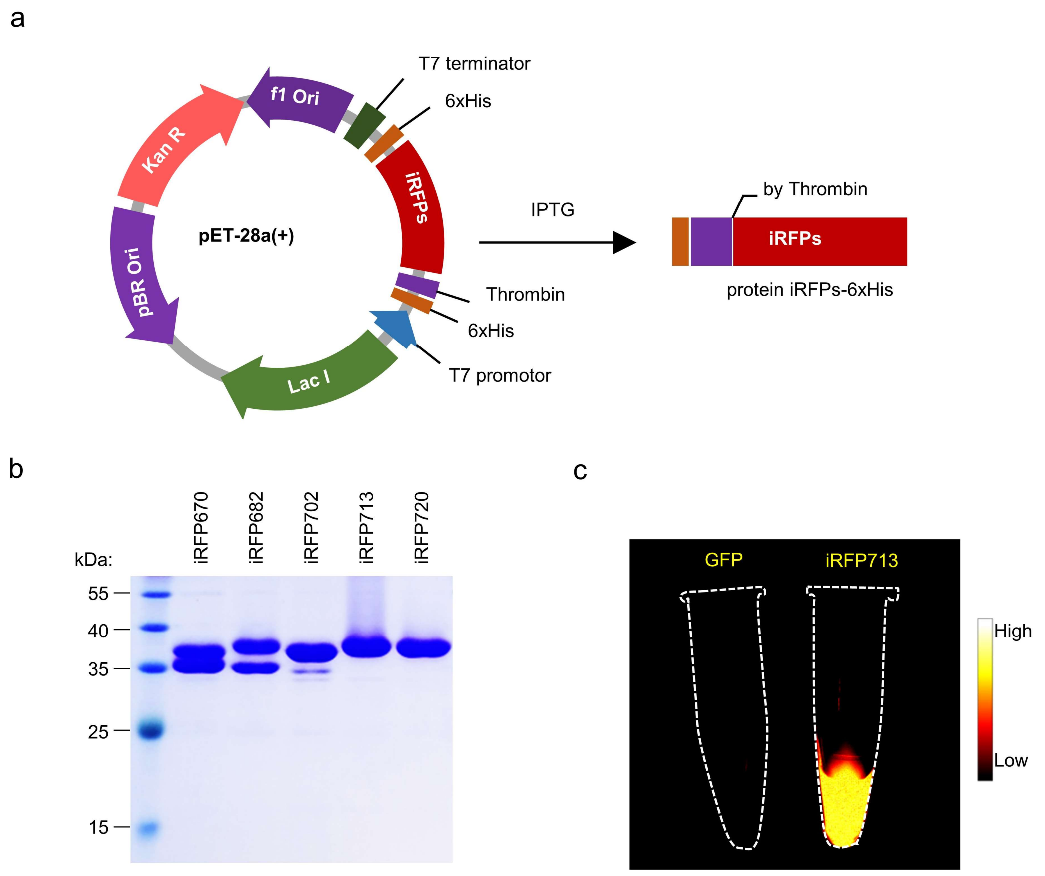

(**a**) Diagram of the recombinant pET28a-iRFPs-6xHis plasmid, for expression of N-terminally 6xHis-tagged proteins with a thrombin site. (**b**) Coomassie blue staining of purified recombinant iRFPs. Experiment was performed in biological triplicate. (**c**) A NIR-II fluorescence image of purified recombinant GFP protein and iRFP713 protein with 690 nm excitation and 900 nm LP filters. Exposure times: 10 ms. Source data are provided as a Source Data file.

Supplementary Figure 2. NIR-II fluorescence imaging of stable iRFP713-expressing cells.

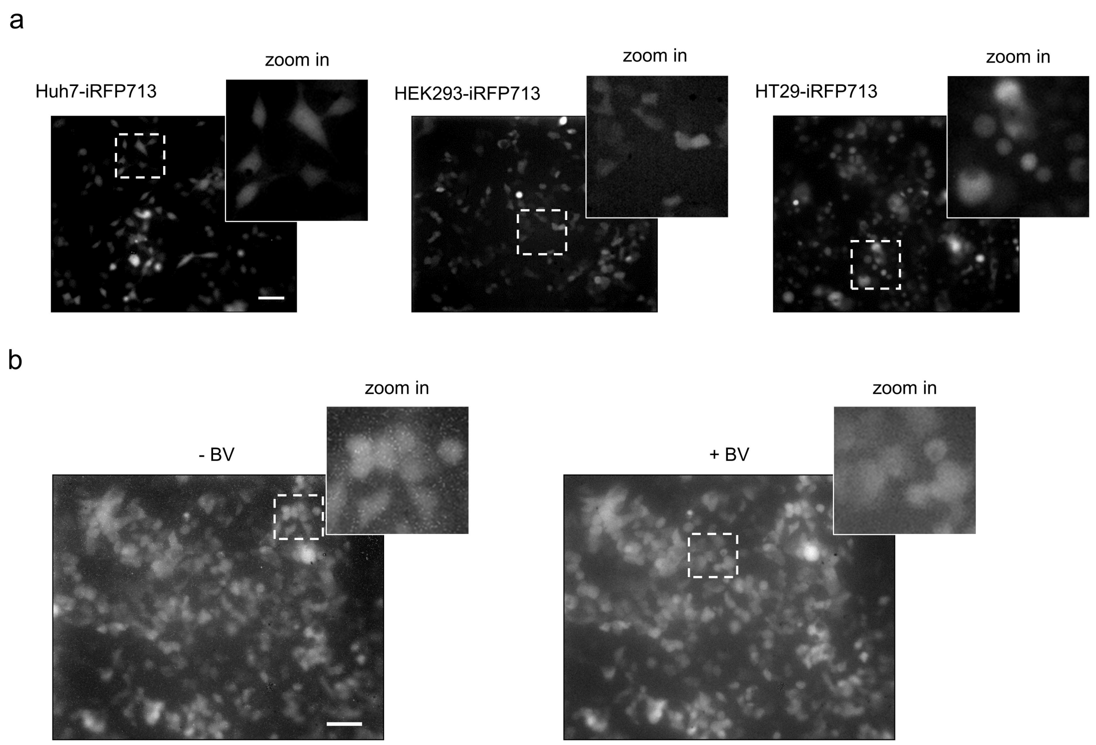

(**a**) NIR-II fluorescence wide-field microscopic images of various iRFP713 stably transfected cells, including Huh7-iRFP713, HEK293-iRFP713, and HT29-iRFP713. (**b**) NIR-II fluorescence images of HEK293-iRFP713 cells without and with addition of 25 μM BV for 2 hours. Each cell experiment was performed three times independently in duplicate each time. Cells were imaged under 690 nm excitation, using 900 nm LP filters. Scale bar: 50 μm.

Supplementary Figure 3. NIR-II fluorescence imaging of mice infected with iRFP713-encoding adenovirus.

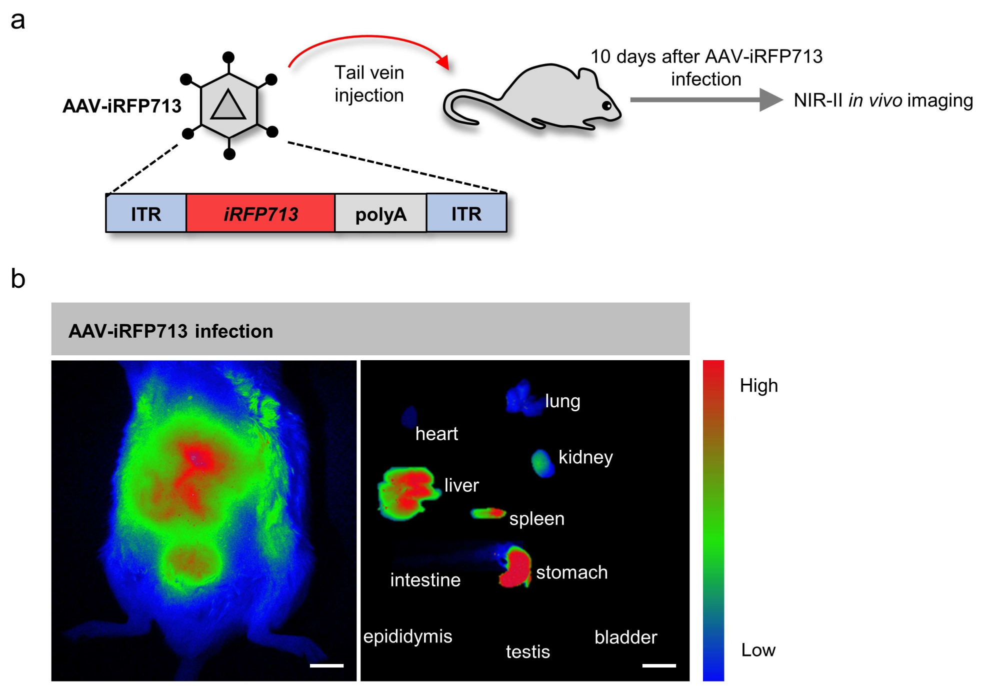

(**a**) Diagram representing the iRFP713-encoding adenovirus (AAV-iRFP713) and the experimental design used in the corresponding animal studies. C57BL/6 mice (male 6-8 weeks old) were intravenously injected with $2x10^9$ infectious units of the iRFP713-encoding adenovirus. (**b**) Ten days after injection, iRFP713-infected mice and their isolated organs were imaged using a 690 nm laser and 900 nm LP filter for excitation and emission, respectively. Exposure times: 50 ms. Scale bars: 2 mm (**b, left**) and 1 cm (**b, right**).

Supplementary Figure 4. Construction of iRFP713 transgenic reporter mice.

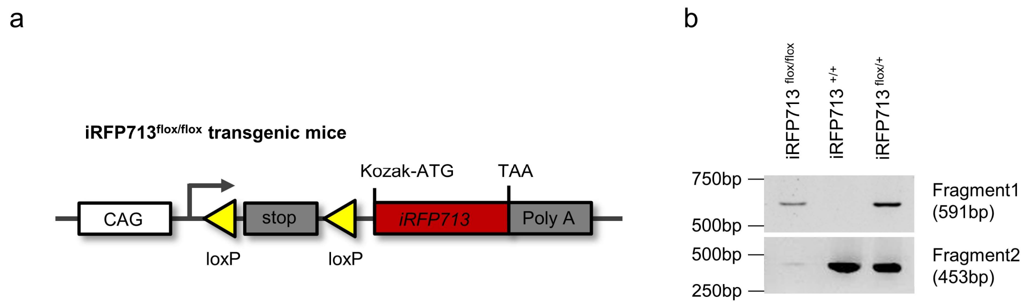

(**a**) Genome structure of the pCAG-loxP-stop-loxP-iRFP713 transgene in iRFP713$^{flox/flox}$ transgenic mice. The transgene consists of a CAG promoter, a loxP-flanked stop gene, followed by iRFP713. Cre-mediated recombination excises the floxed stop gene, resulting in the expression of iRFP713. (**b**) Genotyping PCR of iRFP713 transgenic mice, and independent triplicate experiments were repeated. Source data are provided as a Source Data file.

Supplementary Figure 5. The expression pattern of iRFP713 in different tissues of iRFP713 general expressing mice.

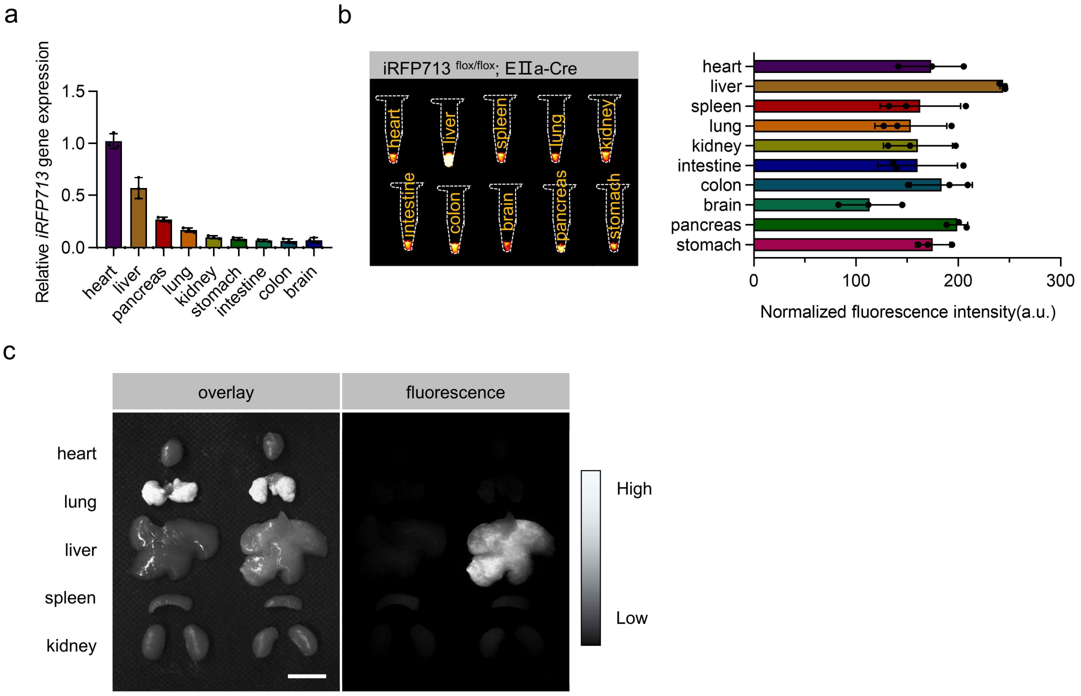

(**a**) *iRFP713* gene expression levels in different tissues of iRFP713$^{flox/flox}$; EIIa-Cre mice (n = 3 mice). (**b**) Representative NIR-II fluorescence images of different protein extracts from the various tissues of iRFP713$^{flox/flox}$; EIIa-Cre mice. Mean fluorescence intensities were normalized to per mg protein of each tissue and are shown on the right (n = 3 mice). (**c**) Representative overlay (NIR-II fluorescence + brightfield channel) and NIR-II fluorescence images of major organs isolated from littermate controls (left) and iRFP713$^{flox/flox}$; EIIa-Cre mice (right). All data presented as mean values ± SEM. Images were acquired using 690 nm excitation and 900 nm LP filters. Exposure times: 30 ms (**b**, **c**). Scale bar: 1 cm.

Supplementary Figure 6. Specific expression of iRFP713 in the liver of hepatocyte-specific iRFP713 expressing mice.

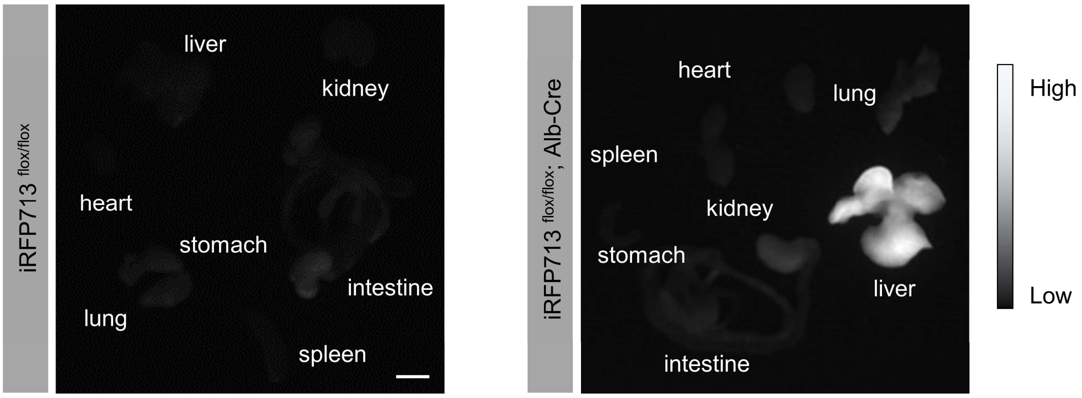

Representative NIR-II fluorescence images of major organs isolated from littermate controls (iRFP713$^{flox/flox}$) and hepatocyte-specific iRFP713 expressing mice (iRFP713$^{flox/flox}$; Alb-Cre). Isolated organs were imaged using 690 nm excitation and 900 nm LP filters. Exposure times: 15 ms. Scale bar: 1 cm.

Supplementary Figure 7. Liver morphology of the hepatocyte-specific iRFP713 expressing mice after partial hepatectomy (PHX).

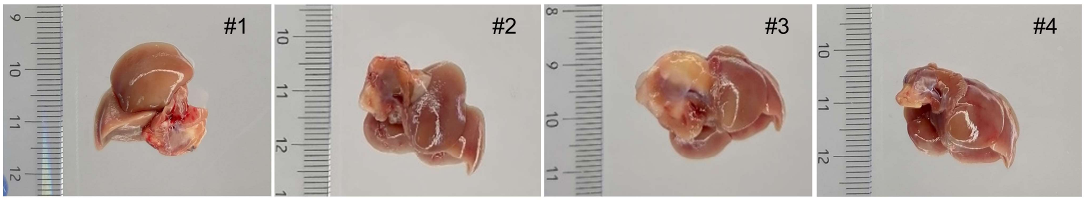

Liver resection model was established by removing the middle lobe and left lobe of iRFP713$^{flox/flox}$; Alb-Cre mice. Ten days after PHX, mice were sacrificed, livers were isolated, and gross liver morphology of each mouse was imaged. The numbers in the upper-right corner of each panel represents individual mice.

Supplementary Figure 8. Effect of iRFP713 expression on hepatocyte proliferation after PHX.

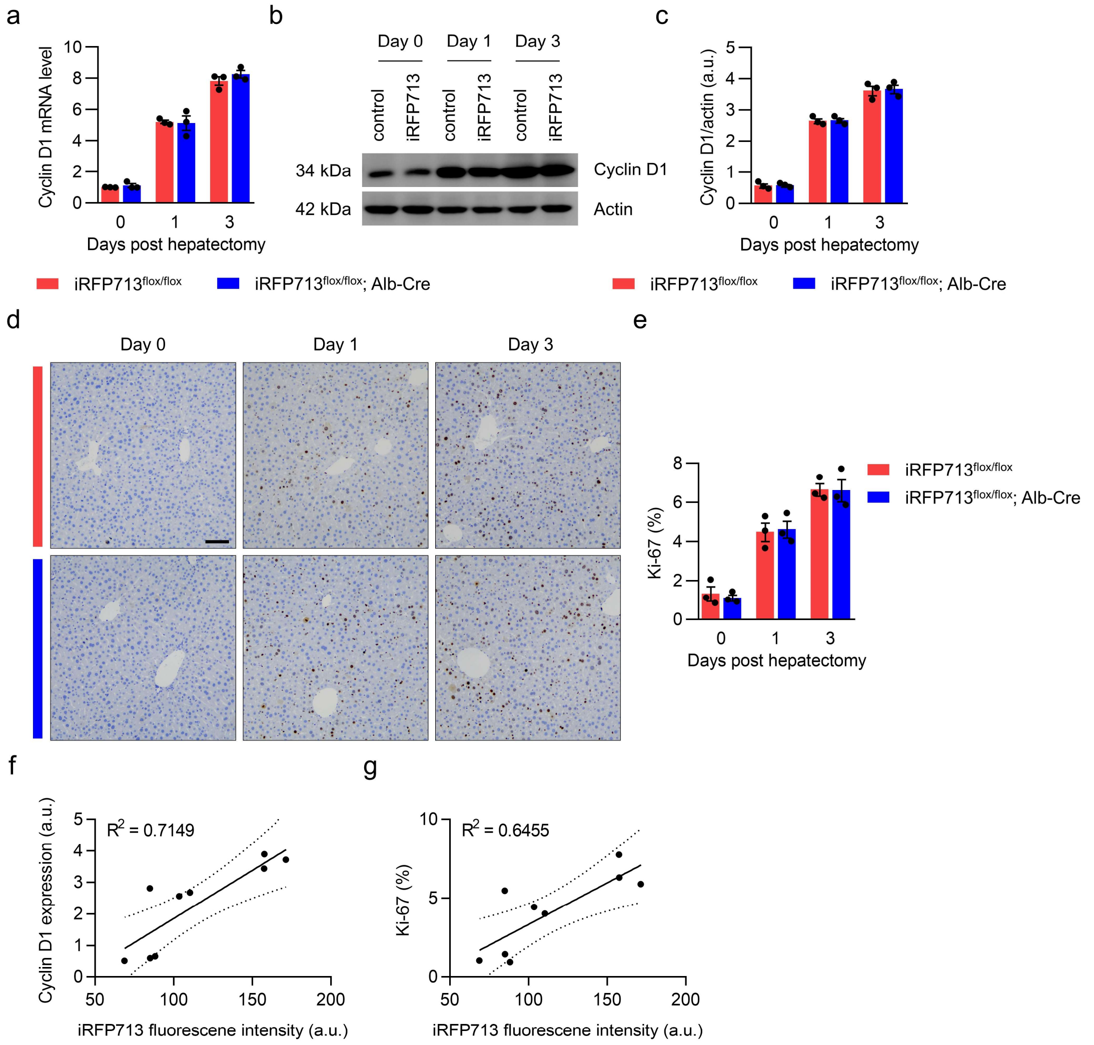

Liver was harvested from iRFP713$^{flox/flox}$ and iRFP713$^{flox/flox}$; Alb-Cre mice at day 0, day 1 and day 3 after PHX. (**a**) mRNA level (**b**) and protein level of cyclin D1, (**c**) intensities of cyclin D1 protein bands were quantified and normalized to actin (n = 3 mice). (**d**) Representative images and (**e**) quantification of IHC staining for Ki-67 (n = 3 mice). The correlations between iRFP713 fluorescence intensity and cyclin D1 protein expression (**f**), and Ki-67 staining (**g**), error bands display 95% confidence intervals. All data presented as mean values ± SEM. Scale bars: 20 μm (**d**). Source data are provided as a Source Data file.

Supplementary Figure 9. Hepatotoxicity and NIR-II fluorescence *in vivo* imaging of ICG.

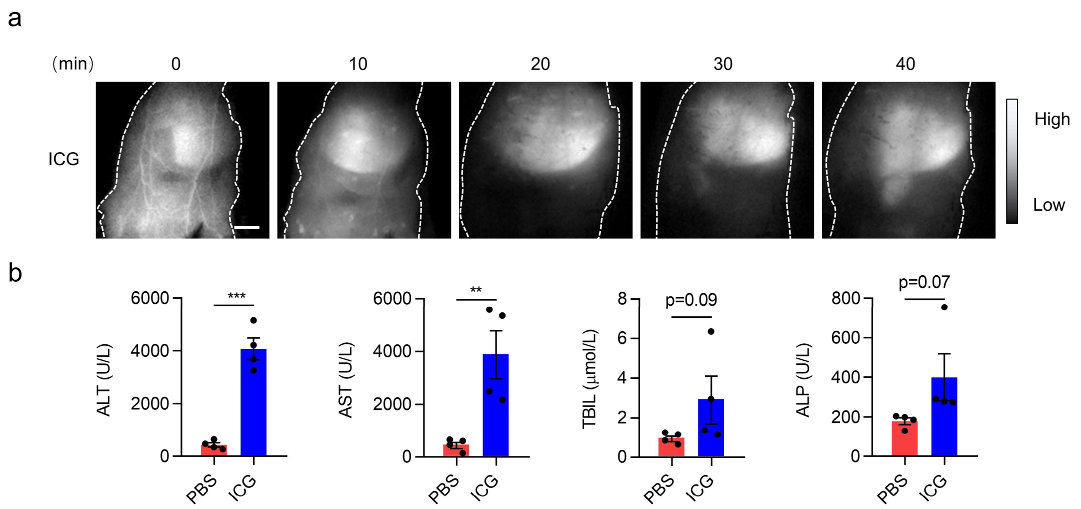

(**a**) Representative fluorescence images of C57BL/6 mice at different time points after intravenous injection of ICG (1 mg/mL). Images were recorded with 793 nm excitation and 900 nm LP collection. (**b**) Four main hepatic function indexes of ICG-treated mice. 6 to 8-week-old C57BL/6 mice underwent liver resection and were subsequently treated with ICG (1 mg/mL) or control PBS. After 24 hours, sera were collected from the mice for liver functional tests, including alanine transaminase (ALT, ***$p<0.001$), aspartate transaminase (AST, **$p<0.01$), total bilirubin (TBIL), and alkaline phosphatase (ALP). Data presented as mean ± SEM; n = 4 mice for each group. Statistical analysis were performed by unpaired two-tailed Student's t test. Exposure times: 15 ms. Scale bar: 5 mm.

Supplementary Figure 10. Comparation of liver proliferation and injury between iRFP713 and ICG.

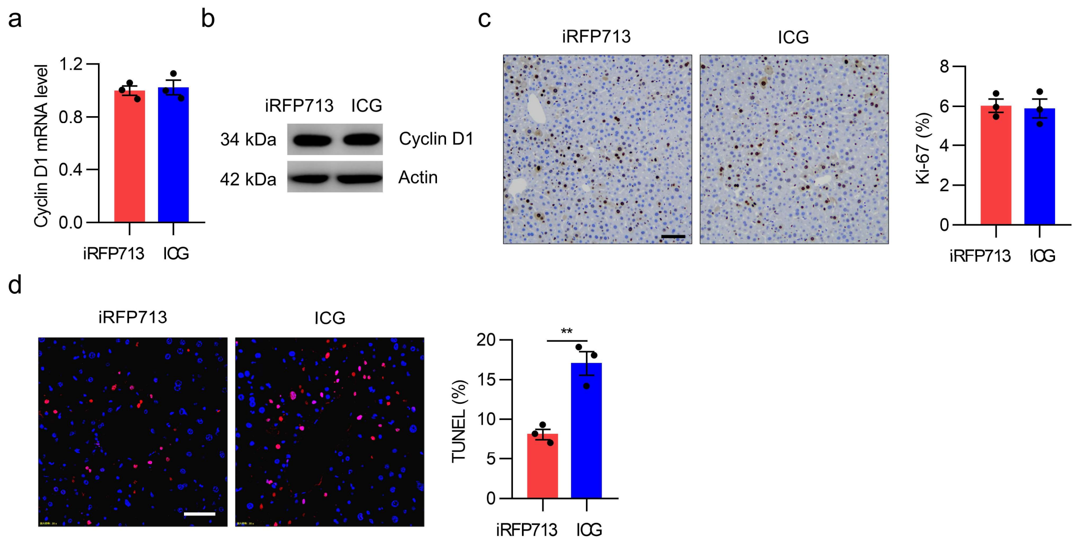

One day after PHX, iRFP713$^{flox/flox}$; Alb-Cre mice and ICG treated C57BL/6 mice were sacrificed, and liver tissues were isolated from the mice for proliferation and injury detection. (**a**) mRNA level and (**b**) protein level of cyclin D1. (**c**) Representative IHC images and quantification of Ki-67 staining on liver sections. (**d**) Representative merged fluorescence images (TUNEL, red and DAPI, blue) and quantification of TUNEL assay (**p<0.01). All data presented as mean ± SEM; n = 3 mice for each group. Statistical analysis were performed by unpaired two-tailed Student's t test. Scale bars: 10 μm (**c**) and 5 μm (**d**). Source data are provided as a Source Data file.

Supplementary Figure 11. Characterization of APAP toxicity model.

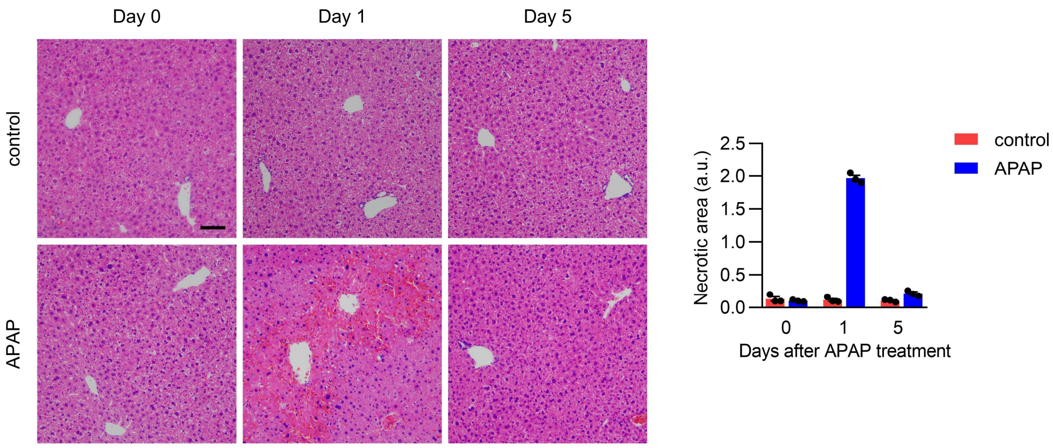

HE staining of liver isolated from iRFP713$^{flox/flox}$; Alb-Cre mice treated with or without APAP (250 mg/kg) at day 0, day 1 and day 5. Quantification of necrotic area was provided in the right. Data presented as mean ± SEM; n = 3 mice for each group. Scale bar: 20 μm.

Supplementary Figure 12. Liver injury and recovery of hepatocyte-specific iRFP713 expressing mice treated with APAP.

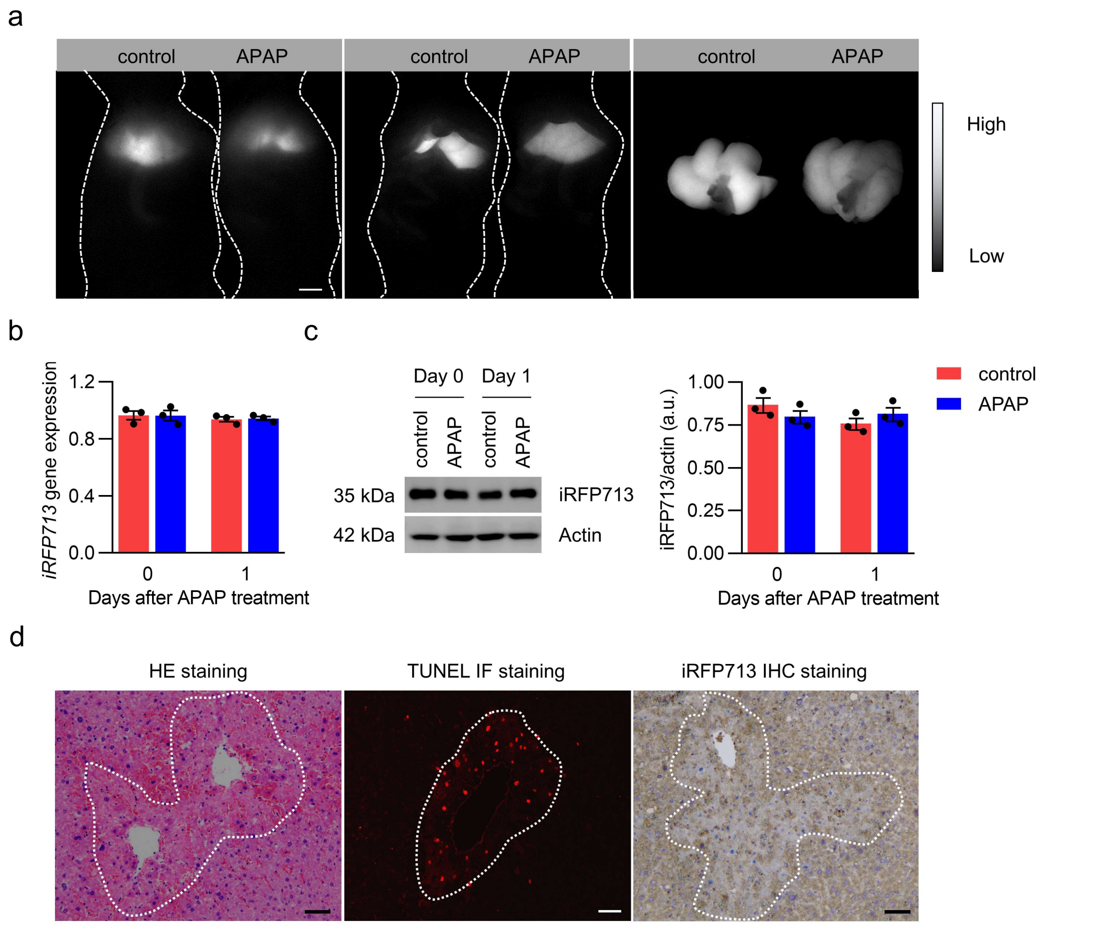

(**a**) Representative fluorescence images of iRFP713$^{flox/flox}$; Alb-Cre mice treated or not APAP. One day after APAP treatment, liver *in vivo* (**left**), liver with laparotomy (**middle**) and isolated liver (**right**) were imaged using 690 nm excitation and 900 nm LP filters. Exposure times: 10 ms (**a, left**), 50 ms (**a, middle**) and 20 ms (**a, right**). (**b**) iRFP713 gene expression level and (**c**) protein level in liver of iRFP713$^{flox/flox}$; Alb-Cre mice at day 0 and day 1 after treated or not APAP. Intensities of iRFP713 protein bands were quantified and normalized to actin (**c, right**). Data presented as mean ± SEM; n = 3 mice for each group. (**d**) Representative HE staining (**left**), TUNEL fluorescence staining (**middle**) and IHC staining of iRFP713 (**right**) in liver sections from iRFP713$^{flox/flox}$; Alb-Cre mice after 1 day of APAP treatment. These staining experiments were repeated independently three times with similar results. Scale bar: 5 mm (**a**), 20 μm (**d: left, right**), and 5 μm (**d: middle**). Source data are provided as a Source Data file.

Supplementary Figure 13. Characterization of multiple low-dose STZ induced diabetes.

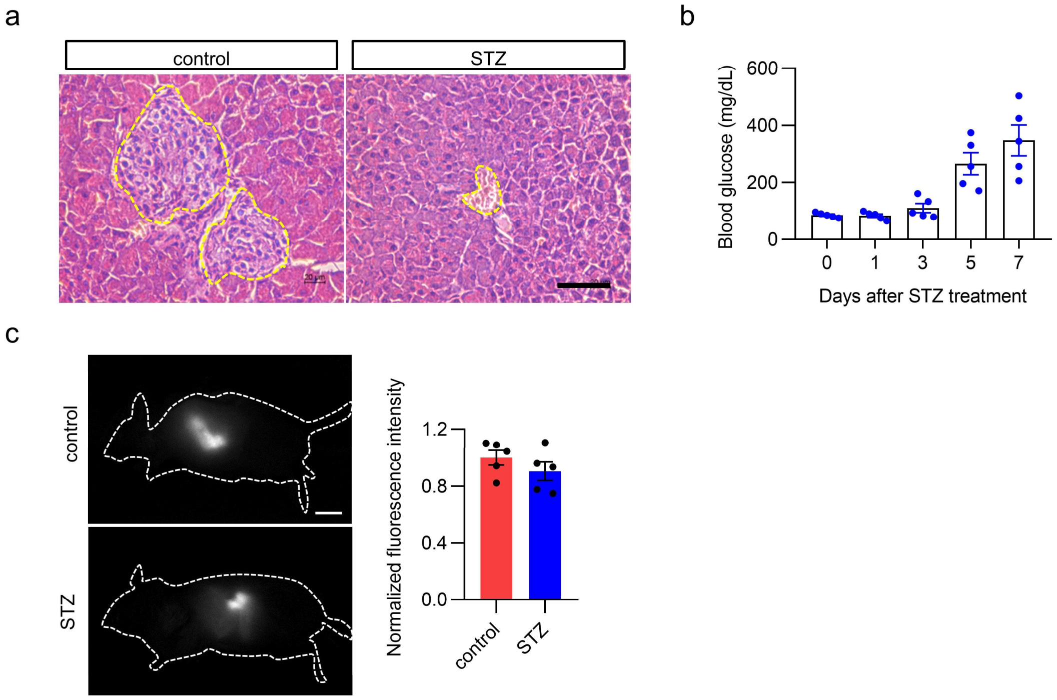

(**a**) Representative photographs of HE stained pancreas isolated from iRFP713$^{flox/flox}$; Pdx1-Cre mice treated with control saline (0.9% NaCl) or multiple low dose STZ (50 mg/kg/day, 5 days). (**b**) Blood glucose records of iRFP713$^{flox/flox}$; Pdx1-Cre mice at different times after STZ treatment. (**c**) Representative NIR-II fluorescence images of iRFP713$^{flox/flox}$; Pdx1-Cre mice treated with control saline or STZ. Mean fluorescence intensity was provided. All data presented as mean ± SEM; n = 5 mice for each group. Images were acquired with 690 nm excitation and 900 nm LP filters. Exposure times: 15 ms. Scale bars: 20 μm (**a**) and 5 mm (**c**).

Supplementary Figure 14. Long-term expression stability of iRFP713 transgenic mice.

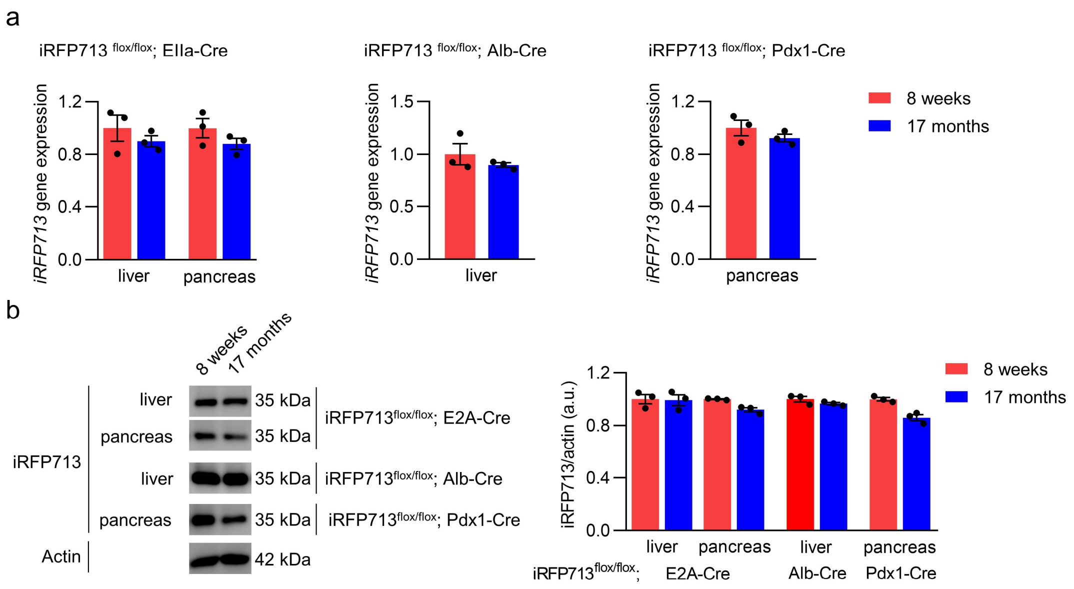

(**a**) Gene expression levels and (**b**) protein levels of iRFP713 in liver or pancreas of different iRFP713 transgenic mice (iRFP713$^{flox/flox}$; EIIa-Cre, iRFP713$^{flox/flox}$; Alb-Cre and iRFP713$^{flox/flox}$; Pdx1-Cre) at weeks 8 and months 17. iRFP713 protein bands intensities were quantified and normalized to actin (**b, right**). Data presented as mean ± SEM; n = 3 mice for each group. Source data are provided as a Source Data file.

Supplementary Figure 15. Comparison of iRFP713 imaging in NIR-I and NIR-II regions.

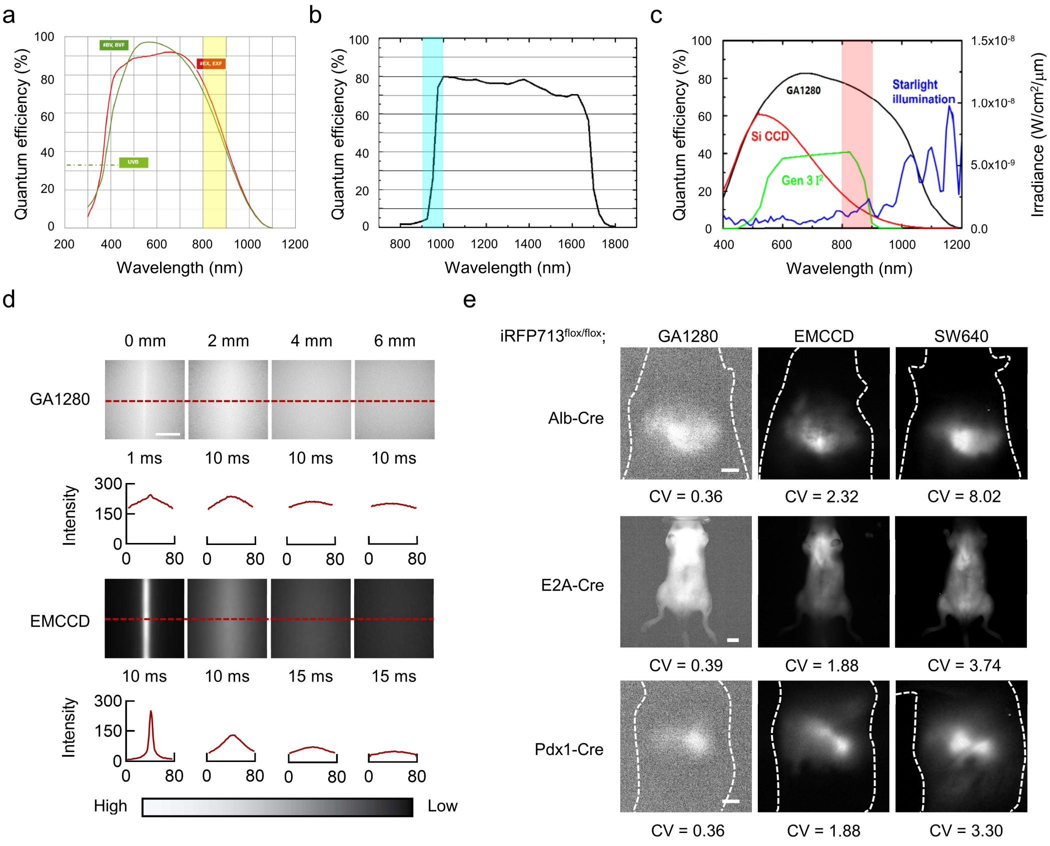

The quantum efficiency curve of (**a**) iXon Ultra 897 camera (EMCCD) and (**b**) SW640 camera (InGaAs FPA). The yellow area indicates the region of 800-900 nm and the blue area indicates the region of 900-1000 nm. (**c**) The quantum efficiency curve of GA1280 camera (vis-NIR 2D-detector, black line). The red area indicates the region of 800-900 nm. (**d**) NIR-I fluorescence (800-900 nm) images of a capillary tube containing iRFP713 immersed at various depths in 1% intralipid (1 mg mL^{-1}, 690 nm Ex). Exposure times: (GA1280) 0 mm: 1 ms; 2 mm, 4 mm and 6 mm: 10 ms. (EMCCD) 0 mm and 2 mm: 10 ms; 4 mm and 6 mm: 15 ms. (**e**) The NIR-I fluorescence (800-900 nm) images using the GA1280 camera (**left**) and iXon Ultra 897 camera (**middle**) and the NIR-II fluorescence (>900 nm) images using the SW640 camera (**right**). (690 nm Ex and 800-900 nm/>900 nm Em). Exposure times: (GA1280) Alb-Cre and Pdx1-Cre: 0.025 ms; E2A-Cre: 0.25 ms. (EMCCD) Alb-Cre, Pdx1-Cre and E2A-Cre: 1 ms. (SW640) Alb-Cre: 5 ms; E2A-Cre: 10 ms; Pdx1-Cre: 1 ms. Scale bars: 5 mm (**d, e**).

Supplementary Table 1. The calculated area SBR values.

**Area SBR values**	**GA1280**	**iXon Ultra 897**	**SW640**
iRFP713$^{flox/flox}$; Alb-Cre	3.07	21.62	213.99
iRFP713$^{flox/flox}$; E2A-Cre	2.33	13.32	72.31
iRFP713$^{flox/flox}$; Pdx1-Cre	2.95	17.83	47.07

Supplementary Table 2. Primers of iRFP713.

**Genotyping primers**	
**Primers**	**Sequence (5'-3')**
iRFP713-F	CACTTGCTCTCCCAAAGTCGCTC
iRFP713-R1	ATACTCCGAGGCGGATCACAA
iRFP713-R2	AGATGTACTGCCAAGTAGGAAAGTC
**RT-qPCR primers**	
**Primers**	**Sequence (5'-3')**
iRFP713-qPCR-F1	CACTCTTCCATCACGCCGAT
iRFP713-qPCR-R1	TGATCGTTTGCCATCACCGA

Supplementary Table 3. Antibodies used for experiments.

**Antibody**	**Catalog Number**	**Company**	**Dilution**
Cyclin D1	60186-1-Ig	Proteintech	1:1000
Ki-67	556003	BD Biosciences	1:500
Actin	4970	Cell signaling technology	1:5000
iRFP713	Not available	HuaAn Biotechnology	1:1000
