# Supplementary material for: Long-term monitoring of intravital biological processes using fluorescent protein-assisted NIR-II imaging
Source: Nat Commun. 2022 Nov 4;13:6643. doi: 10.1038/s41467-022-34274-w (PMC9636246; doi:10.1038/s41467-022-34274-w)
Supplement: Supplementary file 5 — Description of Additional Supplementary Files [file 41467_2022_34274_MOESM5_ESM.pdf]

Title: Supplementary movie 1.
Description: NIR-II fluorescence imaging of newborn whole-body iRFP713-expressing mice and control littermate mice.

Title: Supplementary movie 2.
Description: NIR-II fluorescence imaging of the pancreas-specific iRFP713- expressing mice after laparotomy.
